# Modulation of P2X_4_ receptor activity by ivermectin and 5‐BDBD has no effect on the development of ARPKD in PCK rats

**DOI:** 10.14814/phy2.15510

**Published:** 2022-11-10

**Authors:** Biyang Xu, Oksana Nikolaienko, Vladislav Levchenko, Apurva Swapnil Choubey, Elena Isaeva, Alexander Staruschenko, Oleg Palygin

**Affiliations:** ^1^ Department of Molecular Pharmacology and Physiology University of South Florida Tampa Florida USA; ^2^ Department of Physiology Medical College of Wisconsin Milwaukee Wisconsin USA; ^3^ Bogomoletz Institute of Physiology Department of Cellular Membranology Kyiv Ukraine; ^4^ Hypertension and Kidney Research Center University of South Florida Tampa Florida USA; ^5^ The James A. Haley Veterans Hospital Tampa Florida USA; ^6^ Department of Regenerative Medicine and Cell Biology Medical University of South Carolina Charleston South Carolina USA; ^7^ Division of Nephrology, Department of Medicine Medical University of South Carolina Charleston South Carolina USA

**Keywords:** 5‐BDBD, chronic treatment, ivermectin, kidney, PCK rats, polycystic kidney disease

## Abstract

Autosomal recessive polycystic kidney disease (ARPKD) is an inherited pathology caused mainly by mutations of the polycystic kidney and hepatic disease 1 (*PKHD1*) gene, which usually leads to end‐stage renal disease. Previous studies suggested that the P2X purinoreceptor 4 (P2X_4_R) may play an important role in the progression of ARPKD. To test this hypothesis, we assessed the chronic effects of ivermectin (P2X_4_R allosteric modulator) and 5‐BDBD (P2X_4_R antagonist) on the development of ARPKD in PCK/CrljCrl‐Pkhd1pck/CRL (PCK) rats. Our data indicated that activation of ATP‐mediated P2X_4_R signaling with ivermectin for 6 weeks in high dose (50 mg/L; water supplementation) decreased the total body weight of PCK rats while the heart and kidney weight remained unaffected. Smaller doses of ivermectin (0.5 or 5 mg/L, 6 weeks) or the inhibition of P2X_4_R signaling with 5‐BDBD (18 mg/kg/day, food supplement for 8 weeks) showed no effect on electrolyte balance or the basic physiological parameters. Furthermore, cystic index analysis for kidneys and liver revealed no effect of smaller doses of ivermectin (0.5 or 5 mg/L) and 5‐BDBD on the cyst development of PCK rats. We observed a slight increase in the cystic liver index on high ivermectin dose, possibly due to the cytotoxicity of the drug. In conclusion, this study revealed that pharmacological modulation of P2X_4_R by ivermectin or 5‐BDBD does not affect the development of ARPKD in PCK rats, which may provide insights for future studies on investigating the therapeutic potential of adenosine triphosphate (ATP)‐P2 signaling in PKD diseases.

## Graphical abstract

Recent studies demonstrated the significant involvement of P2X4 and P2X7 receptors in cyst cell calcium signaling. However, neither allosteric modulation nor an antagonism of P2X4 receptors by chronic administration of ivermectin or 5‐BDBD, respectively, affect the progression of ARPKD in PCK rats.

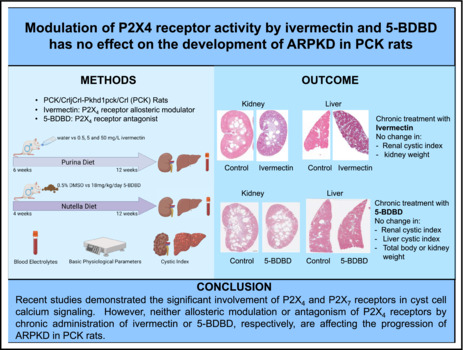

## 1 INTRODUCTION

Autosomal dominant polycystic kidney disease (ADPKD) and autosomal recessive polycystic kidney disease (ARPKD) are two primary forms of monogenic cystic kidney pathology (Bergmann et al., 2018). Unlike ADPKD, which is mainly presented in adults, ARPKD usually occurs perinatally or in early childhood, with a prevalence of 1 in 20,000 live birth (Ilatovskaya et al., 2016, 2019; Sudarikova et al., 2021). Characterized by non‐obstructive fusiform dilations of the renal collecting ducts and ductal plate malformation of the liver, ARPKD is primarily caused by mutations in the polycystic kidney and hepatic disease 1 (*PKDH1*) gene (Turkbey et al., 2009). This gene makes a protein that helps build the bile ducts, and mutations can cause Caroli syndrome, where bile duct stones form in the liver. The recent study also suggested that mutations of DAZ‐interacting protein 1‐like protein (*DZIP1L*) in children promote moderate forms of ARPKD in the absence of mutations in *PKHD1* (Bergmann et al., 2018; Lu et al., 2017).

Various strategies have been used to target the multiple signaling pathways involved in the cyst development of PKDs, for example, vasopressin V2 receptor antagonists, mTOR inhibitors, cAMP inhibitors, etc. (Bergmann et al., 2018; Chang et al., 2011). However, only tolvaptan has been approved as a specific treatment for ADPKD (Gansevoort et al., 2016). More effective therapies are still needed for both ARPKD and ADPKD. Growing evidence suggested that adenosine triphosphate (ATP)‐mediated signaling through the P2X and P2Y receptors may substantially modulate the activity of cystic epithelia and be a potential therapeutic target (Ilatovskaya et al., 2016). A significant increase of extracellular levels of ATP has been detected in primary cultured renal epithelial cells from both ARPKD and ADPKD patients, cyst fluid of ADPKD patients, *cpk/cpk* mice (a murine ARPKD model with a mutation in *cystin* 1 gene), as well as PCK rats (an established model for ARPKD) (Hillman et al., 2004; Palygin et al., 2018; Rangan, 2013; Schwiebert et al., 2000; Wilson et al., 1999). Correspondingly, elevated expression of P2Y_2_, P2Y_6_, and P2X_7_ receptors was detected in cystic kidneys in Han:SPRD (Cy/+) rats (Turner et al., 2004). Our recent study using PCK rats demonstrated shifted profile of P2 receptors with significant involvement of P2X_4_ and/or P2X_7_ receptors in the generation of intracellular Ca^2+^ flux in cystic epithelial cells, while the P2Y component only showed modest contribution (Palygin et al., 2018). Many studies have investigated the therapeutic potential of the P2X_7_ receptor (P2X_7_R) for ARPKD. For example, both P2X_7_R agonists and antagonists have been shown to be capable of modulating the development of renal cysts (Chang et al., 2011; North, 2016). Knockout of P2X_7_R also attenuated cyst growth in PCK rats (Arkhipov et al., 2019). The P2X4 receptor (P2X_4_R) has been linked to epithelial transport in the nephron (Craigie et al., 2013), and many studies have provided evidence for the functional interactions between P2X4 and P2X7 purinergic signaling cascades (Kanellopoulos et al., 2021; Schneider et al., 2017; Trang et al., 2020). Furthermore, recent studies revealed that P2X_1_R, P2X_7_R, and ANG II type 1 receptor (AT_1_R) actions converge at receptor or post‐receptor signaling pathways in ANG II‐dependent hypertension (Kulthinee et al., 2020). However, to the best of our knowledge, no study has tested the effects of P2X_4_R modulators in the setting of any model of PKD.

Here, using PCK rats as a model, we showed that both ivermectin (P2X_4_R allosteric modulator) (Priel & Silberberg, 2004) and 5‐BDBD (P2X_4_R antagonist) (Coddou et al., 2019) did not affect the progression of ARPKD in PCK rats, which may provide insights for future investigation of the therapeutic potential of ATP‐P2 signaling in PKD diseases.

## 2 MATERIALS AND METHODS

### 2.1 Animals and experimental protocols

Animal use and welfare procedures adhered to the National Institute of Health Guide for the Care and Use of Laboratory Animals, following protocols reviewed and approved by the Medical College of Wisconsin Institutional Animal Care and Use Committee. All experiments were carried out in accordance with relevant guidelines and regulations and in compliance with the ARRIVE guidelines (Kilkenny et al., 2010). PCK (PCK/CrljCrl‐Pkhd1pck/Crl) rats were obtained from Charles River Laboratories Inc (Wilmington, MA) and maintained in a standard 12/12 dark/light cycle with water and food (no. 5L0D, LabDiet, St. Louis, MO) provided ad libitum. Animals were randomly selected between the groups, and all experimental protocols were performed simultaneously as the corresponding controls to exclude any variations in cystic formation.

For ivermectin treatment, 0.5, 5, and 50 mg/L ivermectin (# 1260; Tocris/Bio‐Techne, Minneapolis, MN) were delivered to PCK rats in drinking water starting at 6 weeks of age for 42 days, respectively. DMSO was used to dissolve ivermectin to stock concentrations of 10 mM. For oral administration, we used the maximum approved dose of 50 mg/L in our protocol based on the recommendation for rats as described previously (Foletto et al., 2015). Drinking water only was used as a control.

For 5‐BDBD treatment, PCK rats were fed with Nutella dietary supplement, including 18 mg/kg/day 5‐BDBD (# 3579/10; Tocris/Bio‐Techne, Minneapolis, MN) starting at 4 weeks of age for 56 days. Based on the previous reports (Ilatovskaya et al., 2013; Lalo et al., 2014; Palygin et al., 2018; Rasooli‐Nejad et al., 2014), and the maximum dose approved for our protocol, we used 18 mg/kg/day (50 μmol/kg) of 5‐BDBD for 42 days, which we believe is sufficient to give rise to meaningful plasma concentrations. DMSO was used to dissolve 5‐BDBD, and the corresponding control Nutella dietary supplement included 0.5% DMSO. At the end of the experiments, a deep thoracotomy was performed on all anesthetized animals to produce pneumothorax upon the completion of kidney harvest to ensure animal was euthanized.

### 2.2 Biochemical analysis and tissue harvesting

Plasma electrolytes and creatinine were measured with a blood gas and electrolyte analyzer (ABL system 800 Flex, Radiometer, Copenhagen, Denmark). Blood and organ collections were performed at the end of the treatment as previously described (Ilatovskaya et al., 2019; Palygin et al., 2019).

### 2.3 Histochemistry and analysis of cystic index

Animals were anesthetized with isoflurane (5% induction, 1.5% to 2.5% maintenance)/medical grade O_2_, and continually monitored to ensure an adequate level of anesthesia. Laparotomy was performed, and kidneys were flushed with phosphate‐buffered saline (PBS) through the abdominal aorta. Kidneys were extracted, decapsulated, and placed into ice‐cold PBS (Pavlov et al., 2015). A thoracotomy confirms euthanasia. Rat kidneys were formalin‐fixed, paraffin‐embedded, sectioned, and mounted on slides. Briefly, kidney sections were cut at 4 μm, dried, deparaffinized with alcohol, and re‐fixed in Bouin's solution for 1 h at 56°C to improve staining quality. Standard Masson's trichrome staining protocol was applied as previously described (Ilatovskaya et al., 2019; Otali et al., 2016). For ivermectin study, kidney and liver morphologies were assessed by Masson's trichrome staining and scanned by Nikon Super CoolScan 9000 (Nikon). For 5‐BDBD study, kidney and liver morphologies were assessed by Masson's trichrome staining and scanned by Nanozoomer S60 Digital Slide Scanner (Hamamatsu Photonics). Color thresholding method was used to analyze cystic area and fibrosis of kidney and liver samples by using Image J software as previously described (Ilatovskaya et al., 2019). All analyses were conducted in a blind manner.

### 2.4 Statistical analysis

Data are expressed as mean ± SEM and analyzed using GraphPad Prism 9 (GraphPad Software). Different groups were compared using the student's *t*‐test or ANOVA with Tukey post hoc test. *p < 0.05.

## 3 RESULTS

### 3.1 Experimental design and basic physiological parameters of PCK rats

**FIGURE 1:**
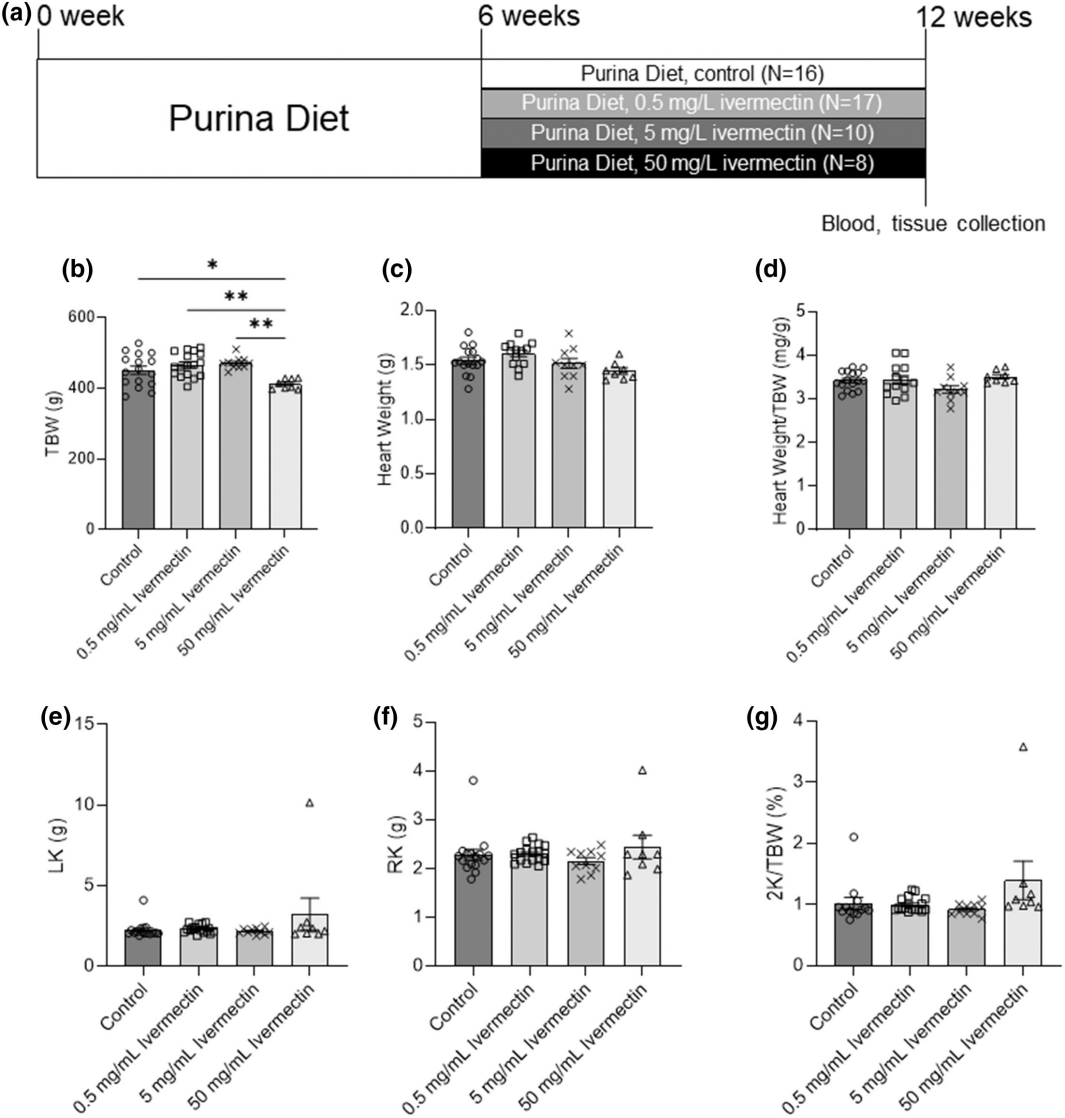
Experimental protocol and basic physiological measurements for PCK rats treated with ivermectin. (a) Experimental protocol showing that 4 groups of PCK male rats were fed a standard Purina 5001 diet and water (control, *N* = 16) or ivermectin in concentrations 0.5 mg/L (*N* = 17), 5 mg/L (*N* = 10), and 50 mg/L (*N* = 8) for 42 days starting at 6 weeks of age. Total body weight (TBW, b), heart weight (c), heart weight to total body weight ratio (Heart Weight/TBW, d), left kidney weight (LK, e), right kidney weight (RK, f), and 2 kidney weight to total body weight ratio (2 K/TBW, %were measured at the end of the protocol, g). Data were expressed as mean ± SEM and compared using one‐way ANOVA followed by a Tukey post hoc test. *p < 0.05, **p < 0.01, ***p < 0.001.

**FIGURE 2:**
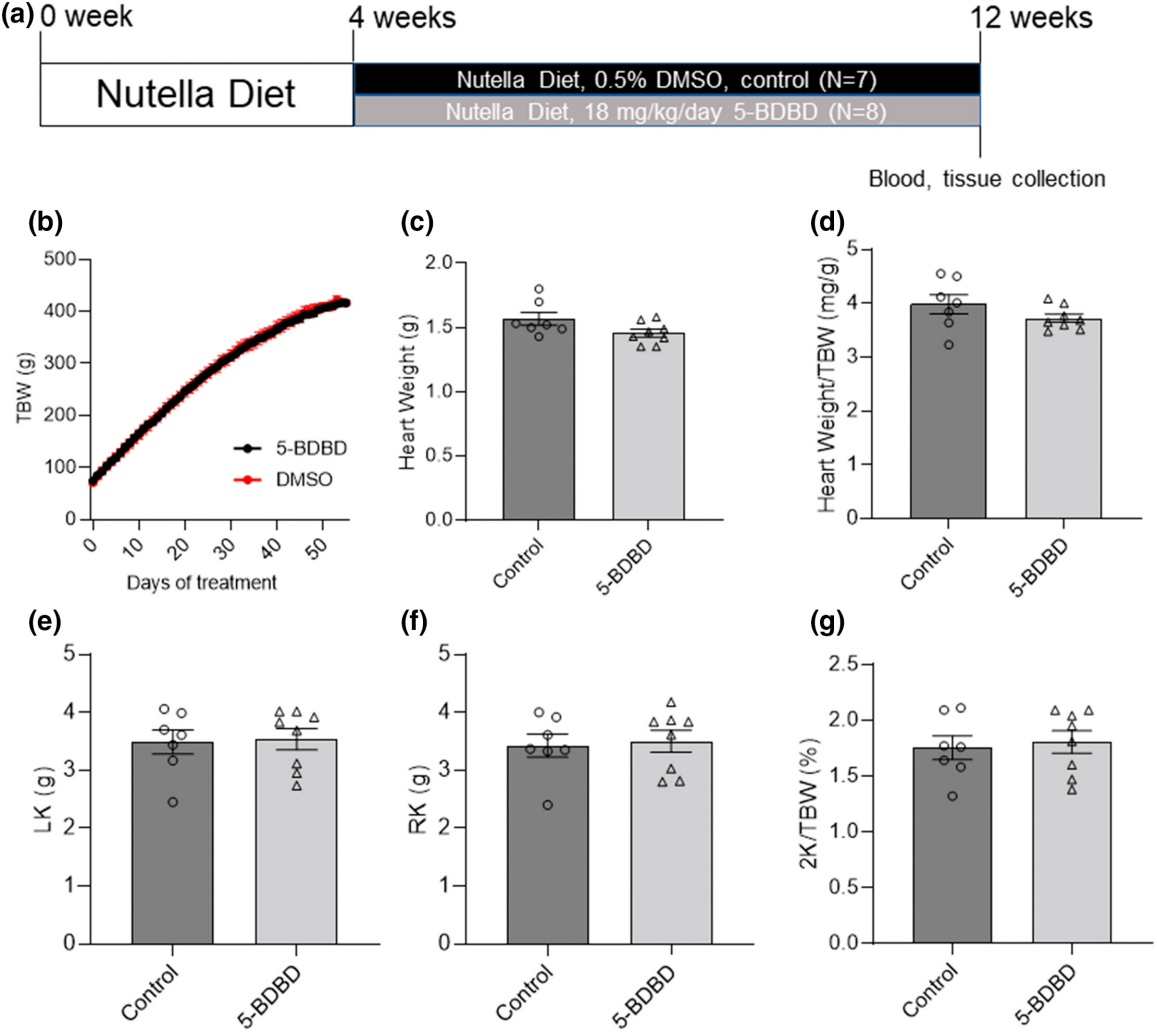
Experimental protocol and basic physiological measurements for PCK rats treated with 5‐BDBD. (a) Experimental protocol showing that the 2 groups of PCK male rats were fed on a Nutella diet with 0.5% DMSO (control, *N* = 7) or 18 mg/kg/day 5‐BDBD (*N* = 8) for 56 days starting at 4 weeks of age. Total body weight (TBW, b) was measured every day. Heart weight (c), heart weight to total body weight ratio (Heart weight/TBW, d), left kidney weight (LK, e), right kidney weight (RK, f), and 2 kidney weight to total body weight ratio (2 K/TBW, %, g) were measured at the end of the protocol. Data were compared by the Student's *t*‐test.

Ivermectin binds to a P2X_4_R and significantly increases purinergic response effectiveness, reducing ATP EC50 values up to ten times (Jelinkova et al., 2006). In vivo, at concentrations 2–3 μM, ivermectin facilitates P2X_4_ receptor activation to ATP (Jelinkova et al., 2006; Priel & Silberberg, 2004). For the ivermectin study, 4 groups of PCK rats on a standard Purina 5001 diet were treated with drinking water only (control, *N* = 16), 0.5 (*N* = 17), 5 (*N* = 10), and 50 mg/L (*N* = 8) ivermectin delivered in drinking water, respectively, starting from 6 weeks of age for 42 days (Figure 1a). For the 5‐BDBD study, 2 groups of PCK rats were fed with standard chow supplemented with Nutella mixed with 0.5% DMSO (control, *N* = 7) or 18 mg/kg/day 5‐BDBD (*N* = 8), respectively, starting from 4 weeks of age for 56 days (Figure 2a). Doses of ivermectin and 5‐BDBD were selected based on the previously published reports (Ahmed et al., 2020; Aryannejad et al., 2021; Coddou et al., 2019; Franklin et al., 2015; Ilatovskaya et al., 2013; Jelinkova et al., 2006; Palygin et al., 2018; Priel & Silberberg, 2004; Srivastava et al., 2020; Wang et al., 2020). To investigate the effect of chronic administration of ivermectin and 5‐BDBD on basic physiological parameters in PCK rats, total body, heart, kidney weights, and blood electrolytes were measured at the end of the protocol. In addition, the effect of ivermectin and 5‐BDBD treatments on cysts development in the liver and kidney was also evaluated at the end of the protocol.

### 3.2 The impact of chronic administration of Ivermectin or 5‐BDBD on PCK rats

Basic physiological measurements indicated that lower doses of ivermectin (0.5 and 5 mg/L) had no significant effects on the body, kidney, and heart weights of PCK rats (Figures 1b–g). Higher doses of ivermectin (50 mg/L) significantly decreased total body weight (TBW; Figure 1b). However, although 50 mg/L ivermectin decreased the TBW of PCK rats, the kidney and heart weights remained unaffected (Figures 1c–g). Similarly, 5‐BDBD treatment had no significant effect on the body, kidney, and heart weights of PCK rats (Figures 2b–g). At the end of the experiments, plasma electrolyte analysis of PCK rats indicated that both ivermectin and 5‐BDBD did not change plasma K^+^, Na^+^, Ca^2+^, Cl^−^, or creatinine levels (Tables 1 and 2).

**TABLE 1:** Blood tests for PCK rats treated with/without ivermectin

	Control (*N* = 16)	0.5 mg/L ivermectin (*N* = 17)	5 mg/L ivermectin (*N* = 10)	50 mg/L ivermectin (*N* = 8)
Potassium (mmol/L)	3.7 ± 0.1	3.7 ± 0.1	3.7 ± 0.1	3.7 ± 0.1
Sodium (mmol/L)	138 ± 1	137 ± 1	139 ± 1	140 ± 1
Calcium (mmol/L)	1.27 ± 0.01	1.25 ± 0.02	1.24 ± 0.01	1.29 ± 0.01
Chloride (mmol/L)	108 ± 1	108 ± 1	108 ± 1	108 ± 1
Creatinine (mg/dl)	0.32 ± 0.02	0.28 ± 0.01	0.30 ± 0.01	0.32 ± 0.03

*Note*: Data were expressed as mean ± SEM.

**TABLE 2:** Blood tests for PCK rats treated with/without 5‐BDBD

	Control (*N* = 7)	5‐BDBD (*N* = 8)
Potassium (mmol/L)	3.5 ± 0.2	3.4 ± 0.1
Sodium (mmol/L)	139 ± 2	140 ± 2
Calcium (mmol/L)	1.32 ± 0.02	1.32 ± 0.01
Chloride (mmol/L)	107 ± 1	108 ± 1
Creatinine (mg/dl)	1.72 ± 0.24	1.64 ± 0.27

*Note*: Data were expressed as mean ± SEM.

### 3.3 The effect of chronic administration of ivermectin and 5‐BDBD on cyst development in PCK rats

**FIGURE 3:**
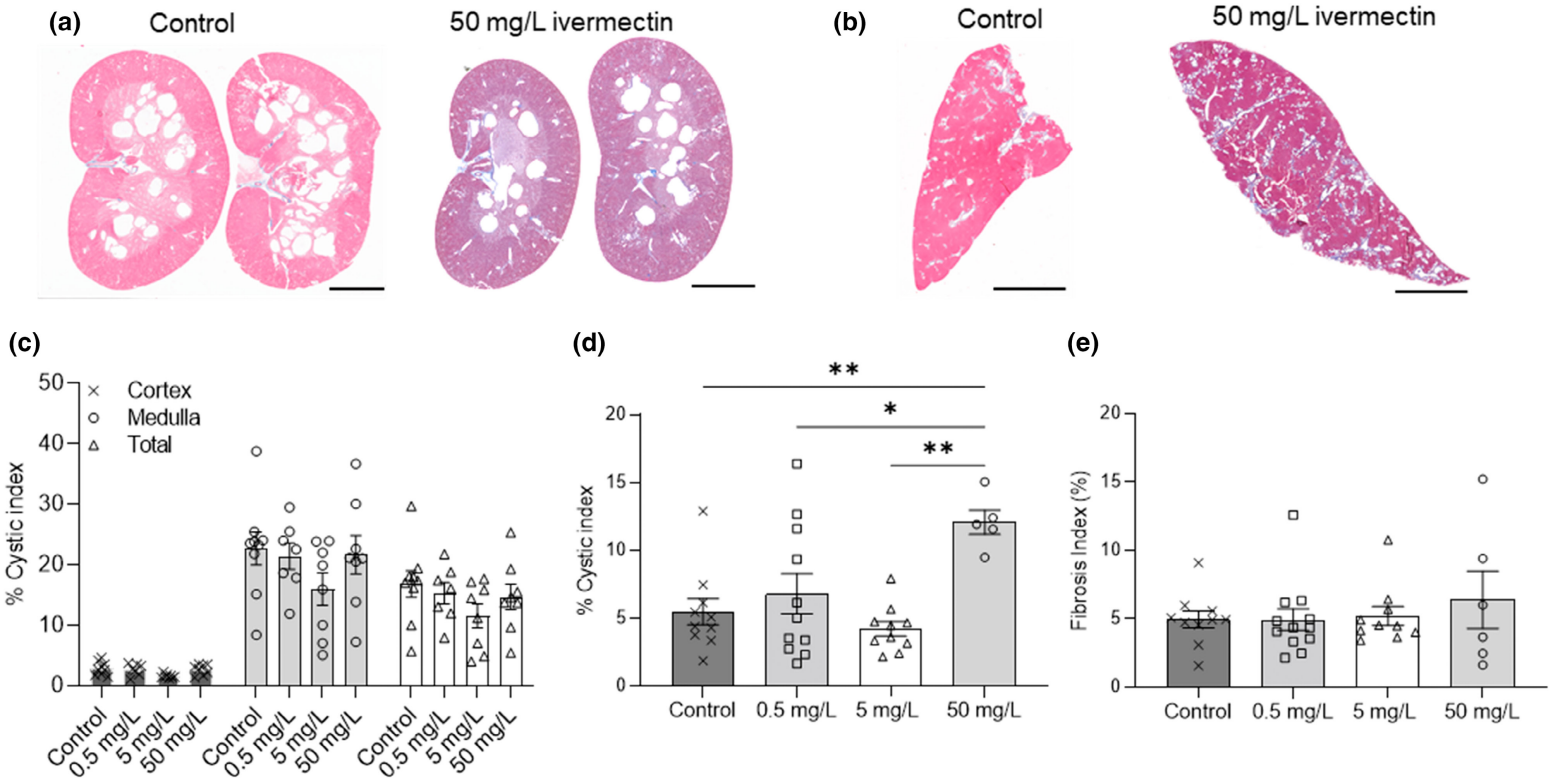
Renal and hepatic cyst development in PCK rats treated with ivermectin. (a, c) Representative images from Masson's trichrome staining for kidney (a) and liver (b) of PCK male rats treated with or without ivermectin. Scale bar: 5 mm. (c, d) Cystic indexes (percentage of cystic area to cortex, medullar or total tissue slice area) of kidney (c) and liver (d) in PCK male rats treated with or without ivermectin. (e) Fibrosis indexes (percentage of fibrosis area to total tissue slice area) of the liver treated with or without ivermectin. Tissues from a minimum of 5 animals per group were analyzed. Data were expressed as mean ± SEM and compared using one‐way ANOVA followed by a Tukey post hoc test. *p < 0.05, **p < 0.01.

**FIGURE 4:**
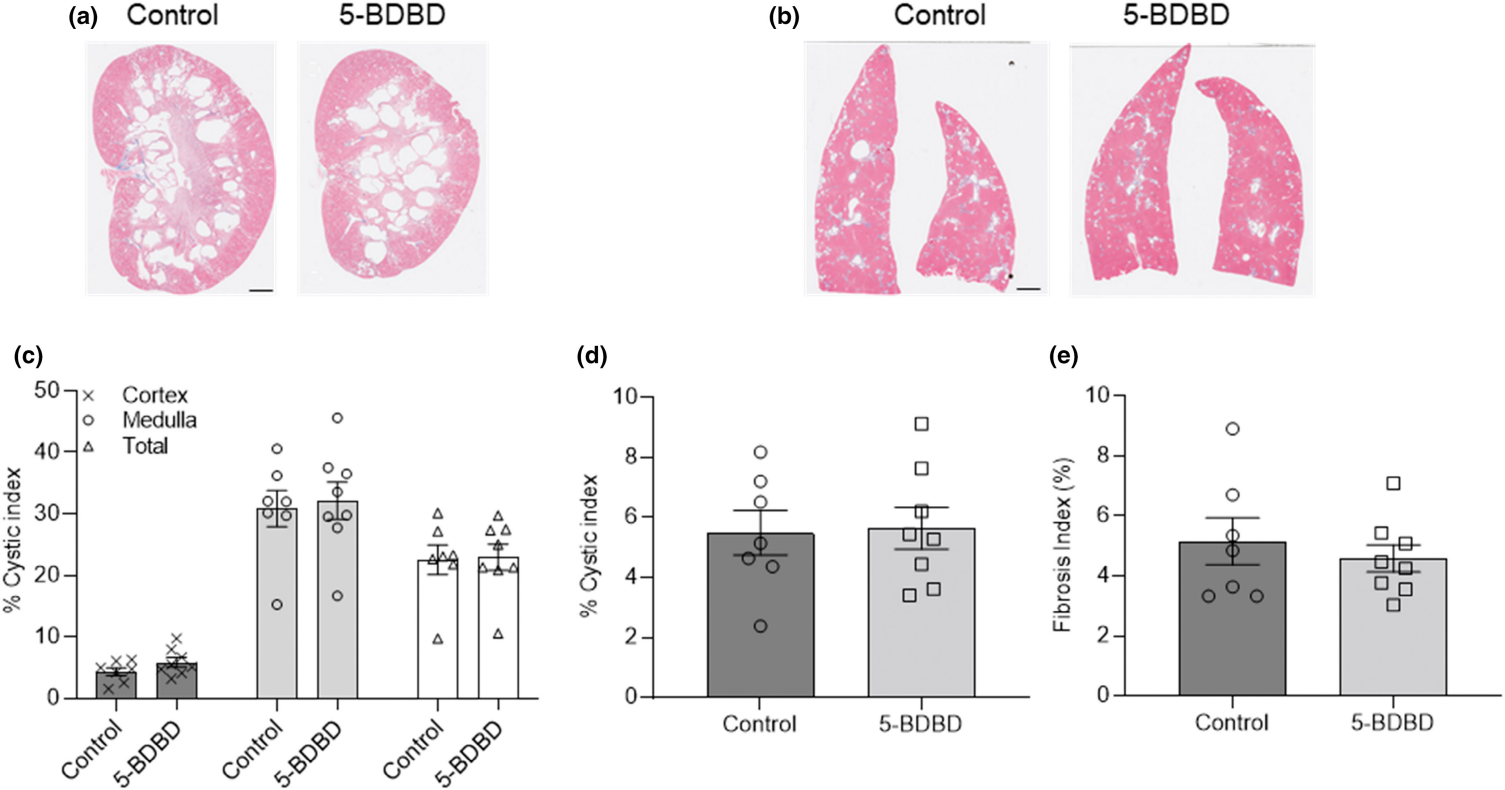
Renal and hepatic cyst development in PCK rats treated with 5‐BDBD. (a, b) Representative images from Masson's trichrome staining for kidney (a) and liver (b) of PCK male rats treated with/without 5‐BDBD. The scale bar (2 mm) is common to all the images. (b, d) Cystic index (percentage of cystic area to cortex, medullar or total tissue slice area) of kidney (c) and liver (d) in PCK male rats treated with or without 5‐BDBD. (e) Fibrosis indexes (percentage of fibrosis area to total tissue slice area) of the liver treated with or without 5‐BDBD. Tissues from a minimum of 7 animals per group were analyzed. Data were expressed as mean ± SEM and compared using one‐way ANOVA followed by a Tukey post hoc test.

Histological examination and cystic analysis of kidney and liver demonstrated similar cystic areas for small doses of ivermectin (0.5 and 5 mg/L) compare to corresponding control groups (Figure 3). Although 50 mg/L ivermectin increased the cystic liver index, the cystic areas in the kidney remain unaffected. Similarly, 5‐BDBD did not affect the cystic area in both the liver and kidney of PCK rats (Figure 4). In addition, the treatment with either 5BDBD or ivermectin did not change the amount of fibrosis in the liver (Figures 3 and 4).

## 4 DISCUSSION

Numerous studies have suggested the role of the ATP‐P2 signaling pathway in PKD diseases (Arkhipov et al., 2019; Arkhipov & Pavlov, 2019; Chang et al., 2011; Ilatovskaya et al., 2016; Monaghan et al., 2021; Schwiebert et al., 2000; Turner et al., 2009; Wilson et al., 1999). Under normal conditions, many kidney cells release extracellular ATP, which is mediated paracrine/autocrine signaling that regulates various aspects of normal vascular and tubular renal physiological functions (Monaghan et al., 2021). Under PKD conditions, abnormally elevated levels of extracellular ATP, released by cystic epithelial cells (CECs), aggravate cyst growth by promoting chloride‐driven fluid secretion, CEC proliferation, and reducing ENaC‐mediated sodium reabsorption from the lumen through P2 receptors (Arkhipov & Pavlov, 2019; Hillman et al., 2004; Rangan, 2013; Schwiebert et al., 2000; Turner et al., 2007). Earlier studies also revealed that the ATP effect on sodium transport was potentiated by ivermectin, which confirmed the involvement of P2X_4_R in this pathway (Zhang et al., 2007).

There are two types of P2 receptors that can be activated by ATP: ionotropic P2X receptors and metabotropic P2Y receptors (Maoumi et al., 2007). Using PCK rats, an established model of ARPKD, our previous study revealed that P2X_4_R and/or P2X_7_R might play a critical role in ATP signaling in cystic epithelial (Palygin et al., 2018). Unlike many studies focusing on the therapeutic potential of P2X_7_R, to the best of our knowledge, no study has investigated the effect of activation of P2X_4_R signaling on cyst development of ARPKD. Here, we investigated the impact of the stimulation of P2X_4_R signaling by ivermectin or antagonism of this pathway by 5‐BDBD on cyst development in PCK rats.

As a widely used antiparasitic drug, ivermectin has been demonstrated to have an allosteric effect on both rat and human P2X_4_R channels (Khakh et al., 1999; Priel & Silberberg, 2004; Syed & Kennedy, 2012). Besides P2X_4_ receptors, ivermectin can also activate GABA_A_, nicotinic acetylcholine, and glycine receptors, although those are not the focus of this manuscript, and no study has suggested the interaction of these receptors with the development of ARPKD (Collins & Millar, 2010; Estrada‐Mondragon & Lynch, 2015; Lynagh et al., 2011). Ivermectin has also become a focus of recent studies due to its proposed potential antiviral activity against COVID‐19 (Formiga et al., 2021). Basic physiological measurements indicated that both 0.5 and 5 mg/L ivermectin did not affect the total body, heart, and kidney weights, suggesting that ivermectin had no side effect on PCK rats under these two concentrations (Figure 1 and Table 1). Indeed, in our experiments, high doses of ivermectin 50 mg/L decreased the total body weight of PCK rats. However, the heart and kidney weights, plasma electrolytes, and creatinine levels of PCK rats remained similar to the control group, indicating the absence of cardiorenal changes associated with long‐term use of the drug. High doses of ivermectin (50 mg/L) increased liver but not cystic kidney index, possibly suggesting that enhanced P2X_4_R activity causes little aggravation of the progression of ARPKD (Figure 3). We speculate that this increase may be promoted by the drug cytotoxicity and negative impact on liver function. In addition, we did not observe any significant differences in the polycystic disease progression between low doses ivermectin‐treated and control rats. Thus, we conclude that the overall effect of drug on the changes in renal function was negligible. This observation is similar to the previously published data used BzATP, and the corresponding activation of the P2X_7_R signaling pathway resulted in the absence of any effects on the cyst formation in the zebrafish model of ADPKD (Chang et al., 2011). Interestingly, another study using ex vivo cultured kidneys from 3‐week‐old *cpk/cpk* mice showed that BzATP reduced cytogenesis (Hillman et al., 2004). The exposure of *cpk/cpk* kidney cells to BzATP reduced cyst number but not the mean cyst area, which can be abrogated by P2X_7_R inhibitor oxidized ATP (OxATP). The contradictory results of mentioned studies indicate that P2X receptor pathway activation and consecutive intracellular Ca^2+^ release may vary in the different models depending on the treatment protocol timeline or possible compensatory expression mechanisms (Hillman et al., 2004; Rangan, 2013; Schwiebert et al., 2000). The latter was established for P2X_4_R in response to P2X_7_R knockout and may suggest that a successful pharmacological strategy required simultaneous targeting of both receptors (Weinhold et al., 2010). On the contrary, the fact that dose‐dependent ivermectin applications did not result in any renal effect makes the possible use of P2X_4_ signaling activators for treating PKD questionable.

In the opposite pharmacological strategy, the selective antagonist for P2X_4_R, 5‐BDBD has been shown to protect against various diseases caused by ATP‐P2X_4_R signaling (Balazs et al., 2013; Chen et al., 2013; Coddou et al., 2019; Srivastava et al., 2020; Syed & Kennedy, 2012). For example, acute P2X_4_R inhibition by oral administration of 5‐BDBD in C57BL/6 mice can protect against ischemic injury at acute and chronic time points after stroke (Srivastava et al., 2020). 5‐BDBD can also inhibit ATP‐P2X_4_R‐mediated NLRP3 inflammasome activation in tubulointerstitial inflammation in diabetic nephropathy (Chen et al., 2013). In our experiments, chronic exposure to 5‐BDBD showed no side effects on PCK rats considering the total body, heart, kidney weights, plasma electrolyte, and creatinine (Figure 2 and Table 2). It also did not affect the cystic indexes of kidneys or liver (Figure 4). Although no study has investigated the effect of P2X_4_R antagonists on ARPKD, one study showed that knocking out P2X_7_R in PCK rats slowed the cyst's growth but not the formation of new cysts, increased epithelial sodium channel activity, and restored impaired channel function (Arkhipov et al., 2019). Our previous study proved that 5‐BDBD could significantly decrease the ATP‐P2X_4_R‐mediated intracellular Ca^2+^ release in freshly isolated kidney cysts (Palygin et al., 2018). The fact that inhibition of P2X_4_R using 5‐BDBD showed no effect in the cyst development of PCK rats in the current study may suggest that although 5‐BDBD showed effect in vitro, inhibition of P2X_4_R may not be sufficient for improving cyst development in vivo. However, this fact does not exclude the possibility of P2X_4_R inhibition in combination therapy with P2X_7_R blockers or in combination with other drugs like Tolvaptan and pasireotide (Hopp et al., 2015).

Growing interests have been focused on the pharmacological therapeutic potential of ATP‐P2 signaling in PKD diseases in the past few years. Our results indicate that targeting P2X_4_R alone did not alter the development of ARPKD. As was recently suggested, P2X_4_ and P2X_7_ may form heterotrimers or homotrimers, and thus P2X_4_/P2X_7_ receptors could potentially be expressed in the plasma membrane of cystic epithelial cells (Kanellopoulos et al., 2021; Schneider et al., 2017; Trang et al., 2020). It may still be worth investigating the effect of other P2X_4_R selective modulators, like psb‐12,054 (inhibitor) or bx‐430 (allosteric antagonist), in combination therapy with corresponding P2X_7_R pharmacology (Ase et al., 2015; Hernandez‐Olmos et al., 2012; Hopp et al., 2015). It should be noted that the current study did not explore possible gender differences in PCK strain. It was reported that PKD is more severe in males than in female PCK rats (Lager et al., 2001). Moreover, intracellular Ca^2+^ signaling and ATP‐induced response are enhanced in male epithelial cells, further driving cell proliferation and cyst growth (Talbi et al., 2021). Thus in our case, the potential difference in treatment between males in females is doubtful, and we did not explore it in our study. Another possible limitation in our study could be related to the drug delivery methods. In our case, the most accessible oral‐based treatment provides low effectiveness, and future studies may use osmotic minipumps or other more effective drug delivery to the renal tissue. Besides the lack of severe side effects of chronic ivermectin and 5‐BDBD exposure, our study provides additional insights into the therapeutic potential of these compounds for other applications (Khir et al., 2021; Khoja et al., 2016; Montilla et al., 2020; Ulmann et al., 2013; Varma et al., 2009; Zhang et al., 2020).

### 4.1 Perspectives and significance

In conclusion, this study revealed that modulation of P2X_4_R signaling by ivermectin (P2X_4_R‐potentiating drug) or 5‐BDBD (P2X_4_R antagonist) does not affect the development of ARPKD in PCK rats, which may provide insights for future studies on investigating the therapeutic potential of adenosine triphosphate (ATP)‐P2 signaling in PKD diseases.

## AUTHOR CONTRIBUTIONS

OP and AS conceived the study. ON, VL, EI, and OP provided investigation. BX, ON, ASC, and OL analyzed the data. BX wrote the original draft of the manuscript. BX, ON, ASC, EI, VL, AS, and OP reviewed and edited the manuscript. All authors approved the final version of the manuscript.

## FUNDING INFORMATION

This research was supported by the National Institutes of Health grants R01 DK126720 (to OP), R35 HL135749 (to AS), Department of Veteran Affairs grant I01 BX004024 (to AS), endowed funds from the SC SmartState Centers of Excellence (to OP).

## DISCLOSURES

None declared.

## ETHICS STATEMENT

The Institutional Animal Care and Use Committee (IACUC) of the Medical College of Wisconsin gave their approval to perform this study on 5/23/2017, with the reference number AUA1061. The Medical College of Wisconsin IACUC guarantees that institutional guidelines and regulations are followed and that all adverse events are reported to the IACUC as soon as possible.
